# Food Safety Practice and Its Associated Factors among Meat Handlers in North Shewa Zone, Oromia, Ethiopia

**DOI:** 10.1155/2022/5829352

**Published:** 2022-08-18

**Authors:** Samuel Chane Teferi

**Affiliations:** Department of Biology, Salale University, P.O. Box. 245, Fiche, Ethiopia

## Abstract

**Background:**

Foodborne illness is one of the major public health problems globally. The majority of foodborne diseases arise from foods of animal origin. Hence, this study was proposed to evaluate meat handling practices and associated factors working in butcher shops in North Shewa Zone.

**Methods:**

Data were collected through face-to-face interviews using a pretested structured questionnaire. Data were entered into a computer and analyzed using SPSS version 26. Binary logistic regression was used to identify factors associated with meat handling practice.

**Result:**

The majority, 128 (57.1%), of meat handlers smoke in workplaces, and 20 (8.9%) of meat handlers handled money while processing meat. 180 (80.4%) of meat handlers process/handle meat when they had cuts, wounds, bruises, or injuries on their hands. 12.1% and 15.6% of meat handlers took food safety training and medical checkups, respectively. 51.3% of meat handlers had good meat handling practices. Knowledge (adjusted odds ratio [AOR] 2.99, 95% confidence interval [CI] 1.73-5.15), attitude (AOR = 1.94, 95% CI: 1.12-3.37), experience (AOR = 4.27, 95% CI: 2.34-9.85), medical checkup (AOR = 3.87, 95% CI: 1.67-8.96), and educational status (AOR = 5.50, 95% CI: 1.05-28.75) were significantly associated with meat handling practices.

**Conclusions:**

Food hygiene training before employment and awareness creation for meat handlers should be strengthened. Routine inspections by responsible authorities are also recommended. Future studies should focus on the enumeration of bacterial load from utensils and meat handlers.

## 1. Introduction

Foodborne illness is one of the major public health problems globally [1, 2]. According to the Centers for Disease Control and Prevention, foodborne diseases caused approximately 76 million illnesses annually among the United States of America's 290 million residents, as well as 325,000 hospitalizations [3]. The European Food Safety Authority in a report published in 2013 shows that a total of 5,648 foodborne outbreaks were reported in the European Union in 2011, causing 69,553 human cases, 93 deaths, and 7,125 hospitalizations [4].

Foodborne diseases occur commonly in developing countries as a result of poor food handling and sanitation practices, poor food hygiene laws, weak regulatory mechanisms, poor funding to purchase safer equipment, and a lack of education among meat handlers [2, 5]. Moreover, it has also been reported that the majority of foodborne diseases arise from food of animal origin [5]. Sources of contamination during meat processing include the equipment, water, contact surfaces, and personnel [6, 7]. The most important spoilage microorganisms of meat are *Campylobacter*, *Clostridium*, *Listeria*, *Staphylococcus*, *Bacillus*, *Acinetobacter* spp., *Moraxella* spp., *Salmonella*, and *Pseudomonas* spp. [2]. According to Assefa et al. [8], improper food handling and poor personal hygiene of workers contribute to approximately 97% of foodborne disease outbreaks among consumers and have led to death in some cases.

According to Tegegne and Phyo [9], the knowledge and level of training of meat handlers in the meat industry are of particular importance in ensuring the health and safety of the consumer. The different authors reported that food handlers have different food safety knowledge levels, and sometimes, an adequate knowledge level does not translate into good hygienic practices when processing and handling food products [10–12]. The three pillars such as food safety knowledge, attitude, and practice are playing a fundamental role in food poisoning outbreaks prevention and control [13–15]. Practice, knowledge regarding meat safety laws, regulations, and personal hygiene of meat handlers are poor. Researches from Ibadan (South-western Nigeria) [9], Ethiopia [9, 15], Iran [11], South Africa [6, 7], and Malaysia [5] have shown that most meat handlers lack meat safety knowledge and adequate training and are frequently engaged in poor handling practices.

In Ethiopia, meat product and consumption is dramatically increasing from time to time, and the consumption of raw meat becomes a symbol of status [15, 16]. Moreover, meat is sold and exhibited in open shops without proper shielding. Besides, most of the meat products in the butcher shops were held on hangers for an extended period which may give sufficient time for the growth of spoilage/pathogenic microorganisms [3, 9]. There has been no study regarding meat handling practice and its associated factors in the study area. Hence, the present study was proposed to evaluate meat handling practices and associated factors working in butcher shops in North Shewa Zone, Oromia, Ethiopia. Due to budget constraints, bacteriological analysis from meat handlers and utensils was not done.

## 2. Methods

### 2.1. Study Design, Setting, and Period

The study was conducted in selected districts/woreda of the North Shewa Zone. The zone is bordered on the south by Addis Ababa on the southwest by West Shewa, on the north by the Amhara Region, and on the southeast by East Shewa. Based on the Central Statistical Agency of Ethiopia projection in 2017, this zone has a total population of 1,870,687 of which 933,273 are males and 937,414 are females, with an area of 10,322.48 square kilometers. A total of 314,089 households were counted in this zone, which results in an average of 4.56 persons to a household and 303,609 housing units. The main economic activity of the zone is agriculture, the zone is gifted with livestock. A cross-sectional study was conducted to evaluate meat handling practices and associated factors among meat handlers working in butcher shops in three selected districts of North Shewa Zone from May to August 2021.

### 2.2. Inclusion and Exclusion Criteria

Meat handlers working in selected butcher shops who have direct contact with meat (waiter, cooker, and meat cutter) were considered as a study population. Those meat handlers who are ill at the time of data collection were excluded from the study.

### 2.3. Sample Size Determination and Sampling Procedures

The sample size (*n*) was determined through a single population proportion formula by taking the proportion (*P*) of 50% with poor practice considered, and there was no previous investigation in the study area, a significance level of 5% (*α* = 0.05), Z*α*/2 = 1.96, and the margin of error 5% (*d* = 0.05).

(1) $$\begin{array}{c} n={\left(\frac{\text{Z}\alpha }{2}\right)}^{2}\frac{P\;\left(1-P\right)}{d^{2}\;},\\ n={\left(1.96\right)}^{2}\times \frac{0.5\;\left(1-0.5\right)}{{\left(0.05\right)}^{2}}=384. \end{array}$$

Since the total number of the source population was below 10,000, a correction formula was used to calculate the final sample size. By adding a 10% nonresponse rate, the calculated sample size for this study was 224. From the total woreda/districts found in the zone, three districts, namely Grar Jarso (which includes the administrative city of the zone, Fiche town), Gerba Guracha/Kuyu, and Debre Libanos, were purposively selected depending on the number of butcher shops available during data collection. A random sampling technique was used to select butcher shops and meat handlers in each woreda.

### 2.4. Data Collection Tools and Procedures

Data were collected through face-to-face interviews using a pretested structured questionnaire. Data were collected by trained data collectors. The questionnaire was first prepared in English language and translated to local languages and back to English to check for consistency. The pretest was performed on 5% of meat handlers from other districts found in the zone, and then correction and amendment were undertaken based on the gaps identified. The questionnaire used for data collection was adopted from similar literature after critically reviewing published articles [9, 11, 15–19]. The questionnaire comprised of four parts, and these were (I) sociodemographic profile of meat handlers, (II) knowledge of foodborne diseases and proper meat handling related questions, (III) attitude questions related to hygienic/safety measures, and (IV) self-reported practices (includes proper personal protective equipment and personal hygiene). To evaluate the attitude level of meat handlers, respondents were asked 20 questions, and those who scored greater than or equal to 70% (14/20) were considered as having a “good level of attitude,” and those who scored less than 70% (13/20) were considered as having a “poor level of attitude” [9, 15–17]. To evaluate the practices of meat handlers, respondents were asked 20 questions, and those who scored greater than or equal to 70% (14/20) were considered as having a “good level of practice,” and those who scored less than 70% (13/20) were considered as having a “poor level of practices” [15, 17]. To evaluate the level of knowledge, respondents were asked 20 questions, and those who scored greater than or equal to 70% were considered as having a “good level of knowledge” (14/20), and those who scored less than 70% (13/20) were considered as having a “poor level of knowledge” [9, 15–17].

### 2.5. Study Variables

The dependent variable in this study was meat safety practice, and the independent variables were gender, work experience/service year, hygiene training, educational status, age, medical checkup, marital status, income, knowledge, and attitude.

### 2.6. Data Analysis

Consistency and completeness of data were verified during collection, entry, and analysis. Data were entered into a computer and analyzed using SPSS version 26. Variables having a *p* < 0.2 in the bivariable analysis were exported to multivariable logistic regression. Variables that had a significant association with meat handling practices in multivariable analysis were identified based on an adjusted odd ratio (AOR) with a 95% confidence interval (CI) and *p* < 0.05.

### 2.7. Ethical Considerations

During data collection, verbal consent was obtained from study participants after the purpose of the study was explained. Any worker was neither forced to participate against their will nor paid for their participation, and the information they gave was kept confidential.

## 3. Result

### 3.1. Sociodemographic Characteristics of Meat Handlers

Two hundred twenty-four meat handlers responded to the questionnaire, which gives a 100% response rate. Eleven (4.9%) of the respondents could not read and write. 17.8% of meat handlers attend higher education, and the majority of respondents in the present study were males (79.5%). 12.1% and 15.6% of meat handlers took food safety training and medical checkups, respectively (Table 1).

### 3.2. Meat Handling Practices

Of two hundred twenty-four meat handlers, 51.3% had a good level of self-reported practice, 62.9% had a good level of attitude, and 52.2% had a good level of knowledge. The majority, 199 (88.8%), of meat handlers wash their hands after handling waste/garbage, 200 (89.3%) of meat handlers wash their hands after using the toilet, and 198 (88.4%) of meat handlers wash their hands before and after handling meat. 43.8% of meat handlers wore an apron and 48.7% remove personal staff such as rings, necklaces, and watches during work. 20 (8.9%) of meat handlers handled money while processing meat. The majority of the respondents handle/processed meat when they had cuts, wounds, bruises, or injuries on their hands (Table 2).

### 3.3. Factors Associated with the Practice of Meat Handlers

Association of different factors on meat handling practices in multivariable logistic regression analysis showed that educational status (AOR = 5.50, 95% CI: 1.05-28.75), knowledge (AOR = 2.99, 95% CI: 1.73-5.15), attitude (AOR = 1.94, 95% CI: 1.12-3.37), medical checkup (AOR = 3.87, 95% CI: 1.67-8.96), and experience (AOR = 4.27, 95% CI: 2.34-9.85) were found to be significantly associated with meat handling practices with *p* value <0.05 (Table 3).

## 4. Discussion

The study was carried out to assess meat handling practices and associated factors working in butcher shops in North Shewa Zone. In the present study, 51.3% of meat handlers had good meat handling practices. This finding is lower than studies in Gondar (66.4%) [15], Dangila (52.5%) [1], and Dubai (81.74%) [20]. But the present study is higher than studies conducted in Gondar town (49%), Imo State, Nigeria (50%), and Turkey (48.4%) [21–23]. These irregularities might be due to variations in sample size, study period, cutoff points, and sociodemographic conditions of the study subjects. In the present study, the odds of a good level of meat handling practice were 1.94 times higher among those with a good attitude than their counterparts (AOR = 1.94, 95% CI: 1.12-3.37). This finding is supported by previous investigations conducted in public food handling establishments in northwest Ethiopia [14], Malaysian food handlers [24], Gondar meat handlers [15], and Debarq mothers [13]. This difference may be due to the strong linkage between positive attitudes and maintaining safe food handling practices. Thus, a good level of attitude has a key role to decrease the chance of foodborne disease outbreaks and the proximal factor that determines the translation into observable action [16].

Meat handlers with a good level of knowledge were 2.99 times more likely to have good food safety practices than those with a poor level of knowledge (AOR = 2.99, 95% CI: 1.73-5.15). The present study is consistent with Gondar town meat handlers [15] and Eastern Ethiopia [25]. According to Ansari-Lari *et al*. [11] and Grema *et al*. [10], adequate knowledge level does not translate into good hygienic practices when processing and handling food products. Inconsistency in hand washing knowledge and practice occurs in studies conducted among meat handlers in Jigjiga and Gondar town [9, 15] and other countries like Kenya and Tamil Nadu [26, 27]. The odds of a good level of practice among meat handlers who had higher educational status were 5.50 times higher than those who had not written and read (AOR = 5.50, 95% CI: 1.05-28.75). The level of education plays an important part in safeguarding the safety of the consumers because educational level appears to affect the attitude and practice of the participants [28]. In the present study, 60.7% of meat handlers attended secondary and higher education. The present study was supported by a study conducted by Ayaz et al. [29] and Meysenburg *et al*. [30]. The probability of a good level of practice was 3.87 times higher among meat handlers who had medical checkups than those who had not (AOR = 3.87, 95% CI: 1.67-8.96). The present finding is in agreement with Azanaw et al. [21], Chekol *et al*. [14], and Teferi *et al*. [31]. Thus, managers/owners of butcher shops should enforce the requirement of having all meat handlers examined every year for health certificates. The odds of food safety practice were 4.27 times higher among meat handlers who had greater than five years of experience than those who had less than two years' experience (AOR = 4.27, 95% CI: 2.34-9.85). The present finding is supported by a study conducted in food and drink establishments in Fiche town [31]. The possible explanation could be that experienced food handlers may have better knowledge and skills regarding food handling practice.

According to Ansari-Lari et al. [11], knowing the importance of proper handling of meat and proper hand washing is very important since meat handlers can serve as vehicles for cross-contamination and the spread of foodborne pathogens. A finding in Gondar revealed that meat handlers with open skin injury, gastroenteritis, ear or throat diseases, discharging wounds, sores, etc. should refrain from work until they are known not to be harboring dangerous pathogens [15]. In contrast to this, more than half of respondents (53.1%) handle meat while they felt ill in the present study. The majority (89.3%) of respondents in the present study wash their hands after using the toilets. This finding is lower than studies conducted by Soares et al. [17] and Yenealem *et al*. [15] but higher than Tegegne and Phyo [9] and Adesokan and Raji [19]. In the present study, 199 (88.8%) meat handlers wash their hands after handling waste/garbage, and 198 (88.4%) meat handlers wash their hands before and after handling meat. This study is lower than a study conducted by Yenealem et al. [15] and Al-Shabib *et al*. [18], but the present study is higher than a study conducted in Jigjiga town by Tegegne and Phyo [9]. Washing hands by food handlers during processing is considered one key important hygiene practice to prevent cross contamination [11]. In the present study, 73.7%, 57.1%, and 54.9% of meat handlers in the present study eat and drink at workplaces, smoke inside meat processing areas, and wash their hand after sneezing, coughing, and smoking, respectively. Sneezing and handling of money while in food processing and production area may lead to cross-contamination. A higher finding was reported by Al-Shabib et al. [18] and Ansari-Lari *et al*. [11]. Protective clothing helps to protect both the food product and the meat handler from cross-contamination. Moreover, protective clothing should be adequately cleaned and disinfected to eliminate pathogenic microorganisms. In the present study, 73.7% of meat handlers used hairnets, and 43.8% of meat handlers wore a gown. The present study is lower than a study conducted by Al-Shabib et al. [18] and Ansari-Lari *et al*. [11]. The disagreement might be due to the sociodemographic conditions of respondents, development of the researched site, and enforcement capacity of stakeholders. In this study, 51.3% of meat handlers did not remove their stuff such as rings, necklaces, watches, and jewelry while processing meat. The present finding is supported by Yenealem et al. [15] and Tegegne and Phyo [9]. This might be due to some organisms like *Staphylococcus aureus* can build up around those objects and pose a risk for the consumers if they fall on the meat.

## 5. Conclusion and Recommendations

The food safety practices of meat handlers in the present study were unsatisfactory. Educational status, knowledge, medical checkup, experience, and attitude were found to be significantly associated with meat handling practices. Unhygienic meat handling practices, lack of training, regular handling of paper currency, and poor sanitation of the butcher shops are among the main factors identified which compromise the quality of the meat products. Therefore, food hygiene training before employment and awareness creation for meat handlers should be strengthened. This will help the meat handlers to have a better understanding of risks associated with contamination of food with microbiological pathogens and sanitation practices. Routine inspections by responsible authorities are also recommended. Finally, future studies should focus on the enumeration of bacterial load from utensils and meat handlers.

## Figures and Tables

**Table 1:** Sociodemographic characteristics of meat handlers working in butcher shops in North Shewa Zone (*n* = 224).

Variables	Meat handlers	
	Frequency	Percentage
Sex		
Male	178	79.5
Female	46	20.5
Age		
≤27	101	45.8
≥28	123	54.2
Educational status		
Unable to read and write	11	4.9
Primary education (1–8)	77	34.4
Secondary education (9–12)	96	42.9
Higher education (12+)	40	17.8
Marital status		
Single	116	51.8
Married	93	41.5
Divorced	15	6.7
Income		
1000 and below	42	18.8
1001–2000	117	52.2
2001–3000	43	19.2
3001 and above	22	9.8
Year of service (experience)		
<2	95	42.4
3-4	70	31.3
>5	59	26.3
Food safety training		
Yes	27	12.1
No	197	87.9
Medical checkups in the last six months		
Yes	35	15.6
No	189	84.4
Working condition		
Permanent	192	85.7
Contract/daily	32	14.3

**Table 2:** Meat handling practice of meat handlers in butcher shops in North Shewa Zone (*n* = 224).

Practice questions	Response *n* (%)	
	Yes	No
Do you drink or eat at the workplace?	165 (73.7)	59 (26.3)
Do you smoke inside meat processing areas?	128 (57.1)	96 (42.9)
Do you use hand gloves while handling meat?	39 (17.4)	185 (82.6)
Do you handle money while processing meat?	20 (8.9)	204 (91.1)
Do you wash your hands before and after handling meat?	198 (88.4)	26 (11.6)
Do you wash your hands after handling waste/garbage?	199 (88.8)	25 (11.2)
Do you wash your hands after using the toilet?	200 (89.3)	24 (10.7)
Do you wash your hand after sneezing, coughing, or smoking?	123 (54.9)	101 (45.1)
Do you wear a gown while working?	98 (43.8)	126 (56.2)
Do you wash your gowns after each day's work?	27 (12.1)	197 (87.9)
Do you wear a face mask while working?	15 (6.7)	209 (93.3)
Do you wear a hairnet while working?	165 (73.7)	59 (26.3)
Do you polish nails while handling meat?	147 (65.6)	77 (34.4)
Do you properly clean the meat storage area?	180 (80.4)	44 (19.6)
Do you use sanitizer when washing service utensils?	104 (46.4)	120 (53.6)
Do you replace knives or sterilize them after each meat processing?	105 (46.9)	119 (53.1)
Do you remove your work equipment when using the toilets?	151 (67.4)	73 (32.6)
Do you remove your stuff such as rings, necklaces, watches, etc. while processing meat?	109 (48.7)	115 (51.3)
Do you handle/process meat when you are ill?	119 (53.1)	105 (46.9)
Do you handle/process meat when you have cuts, wounds, or bruises on your hands?	180 (80.4)	44 (19.6)

**Table 3:** Multivariable logistic regression of factors associated with the practice of meat handlers working in butcher shops in North Shewa Zone (*n* = 224).

Variables	Meat safety practice		Wald	Sig.	AOR (95% CI)
	Good	Poor			
Attitude					
Good	81	60	5.61	0.018∗	1.94 (1.12, 3.37)
Poor	34	49	1		
Medical checkup					
Yes	27	8	10.00	0.002∗	3.87 (1.67, 8.96)
No	88	101	1		
Knowledge					
Good	75	42	15.57	0.0001∗	2.99 (1.73, 5.15)
Poor	40	67	1		
Experience					
<2 year	36	59	1		
3-4 year	35	35	7.90	0.005∗	2.93 (1.38, 6.21)
>5 year	44	15	18.38	0.0001∗	4.27 (2.34, 9.85)
Educational status					
Not read & write	2	9	1		
1-8	26	51	1.96	0.162	0.58 (0.27, 1.24)
9-12	65	31	4.80	0.028∗	2.39 (1.09, 5.23)
>12	22	18	4.08	0.043∗	5.50 (1.05, 28.75)

∗ Statistically significant at *p* < 0.05.

## Data Availability

The datasets used to support the findings of this study are available from the author upon request.

## Abbreviations

AOR: Adjusted odds ratio
CI: Confidence interval
SPSS: Statistical Package for the Social Sciences.
